# Global research trends on the relationship between IBD and CRC: a bibliometric analysis from 2000 to 2023

**DOI:** 10.1186/s41043-024-00577-5

**Published:** 2024-06-12

**Authors:** Hao Zhang, Huiru Xin, Mengqi Zhao, Chenyang Bi, Yafei Xiao, Yifan Li, Changjiang Qin

**Affiliations:** 1 Department of General Surgery, Huaihe Hospital of Henan University, Kaifeng, 475000 China; 2 Department of Cardiothoracic Surgery, Huaihe Hospital of Henan University, Kaifeng, 475000 China; 3 Department of Gastroenterology, Huaihe Hospital of Henan University, Kaifeng, 475000 China

**Keywords:** IBD, CRC, Bibliometric, CiteSpace, WoSCC

## Abstract

**Objective:**

This study aimed to conduct a bibliometric analysis of research articles on the relationship between inflammatory bowel disease (IBD) and colorectal cancer (CRC) using CiteSpace to summarize the current research status, hotspots, and trends in this field and present the results visually.

**Method:**

Research articles on the relationship between IBD and CRC published from 2000 to 2023 and in English were selected from the Web of Science Core Collection (Woscc) database. The articles were downloaded as “full record and references”. CiteSpace was used to conduct cooperative, cluster, co-citation, and burst analyses.

**Results:**

The literature search revealed 4244 articles; of which, 5 duplicates were removed, resulting in the inclusion of 4239 articles in this study. The United States of America had the highest number of publications, with Mayo Clinic and Harvard University being the most active institutions, and Bas Oldenburg being the most active author. Collaboration among core authors was inadequate. JA Eaden was the most cited author, and CRC was the most common keyword. Burst analysis indicated that Sun Yat-sen University might be one of the institutions with a large contribution to this research field in the future. Cluster analysis showed that earlier research focused more on microsatellite instability, whereas “gut microbiota” and “oxidative stress” are considered current research hotspots and trends.

**Conclusion:**

At present, the primary focus areas of research are “gut microbiota” and “oxidative stress”. With the improvement of healthcare policies and standards, regular endoscopic monitoring of patients with IBD has become an indispensable diagnostic and therapeutic practice. More drugs will be developed to reduce the risk of progression from IBD to CRC. The findings of this study provide valuable insights into the relationship between IBD and CRC for researchers in the same field.

**Supplementary Information:**

The online version contains supplementary material available at 10.1186/s41043-024-00577-5.

## Introduction

Colorectal cancer (CRC) is the third most common cancer and the second leading cause of cancer-related deaths worldwide [1]. By 2035, the number of new CRC cases is projected to increase to 2.5 million worldwide, surpassing the number of gastric and liver cancer cases. In particular, the most significant increase will be observed in developing countries [2]. Notably, a more concerning factor is the decreasing age at the diagnosis of CRC [3]. In recent years, numerous studies have demonstrated the association between the gut environment and genetic factors with CRC, and IBD is considered to play a significant role in the development of CRC [4].

Inflammatory bowel disease (IBD), including Crohn’s disease (CD) and ulcerative colitis (UC), is a chronic, relapsing immune-mediated disease with diverse clinical manifestations and varying severity [5]. Both CD and UC manifest as intestinal inflammation but differ in pathological features, target sites, and clinical presentations. UC primarily affects the colon’s mucosa, with continuous lesions from the rectum upwards, causing diarrhea, abdominal pain, bleeding, and mucus in stools [6]. CD can occur anywhere in the gastrointestinal tract with discontinuous, full-thickness lesions, leading to ulcers, fistulas, and strictures [7].

Over the past few decades, numerous studies have demonstrated the relationship between CRC and IBD. Owing to chronic inflammation of the intestinal mucosa, patients with IBD have an increased risk of CRC [8]. Longer disease duration and extensive UC involvement heighten this risk. Chronic inflammation leads to oxidative stress and DNA damage, activating oncogenes and inactivating tumor suppressor genes, driving the transition from inflammation to cancer, influenced by the host immune response and gut microbiota [9, 10].

To date, few studies have summarized research articles on the relationship between IBD and CRC published over the past two decades, facilitating a better analysis of the current research status and future research trends. Bibliometric analysis, which integrates statistical, metrological, and bioinformatic methods, is used to assess the use, output, and dissemination of literature to identify research trends, academic networks, and scholarly contributions and transform complex data into intuitive visual information [11]. In bibliometric analysis, commonly used metrics include citation count, impact factor, and h-index [12]. These measures aid in quantifying and evaluating the usage, output, and dissemination of literature. Citation count and impact factor gauge the influence and significance of a publication [13], while the h-index reflects a researcher’s impact by taking into account both publication quantity and citation frequency [14]. CiteSpace is a tool used for visualizing and analyzing trends and patterns in scientific literature, facilitating the understanding of research progress in a field of study and the identification and monitoring of research hotspots [15]. To the best of our knowledge, this bibliometric study is the first to conduct a qualitative analysis of research articles on the relationship between IBD and CRC using CiteSpace.

## Data collection and research methodology

The Web of Science (WOS) from Clarivate Analytics is considered one of the leading databases for conducting bibliometric analysis. To minimize potential bias introduced by daily updates, all research articles on the relationship between IBD and CRC were identified and selected exclusively from the Web of Science Core Collection (WoSCC) database [16] on December 11, 2023. The data sources included the Science Citation Index Expanded, Conference Proceedings Citation Index-Science, Conference Proceedings Citation Index-Social Science & Humanities, Book Citation Index-Science, and Book Citation Index-Social Sciences & Humanities, with the publication type being limited to articles. All relevant articles were selected based on titles (T1) and abstracts (AB). The specific keywords used for the literature search are provided in Supplementary Material. The articles included were published between January 1, 2000, and December 1, 2023, and were published in English. The specific inclusion criteria for research articles are detailed in Figure S1 (Supplementary materials).

CiteSpace (6.1R6) was used as the principal bibliometric analysis tool in this study. Developed by Professor Chaomei Chen at Drexel University in the USA, CiteSpace is a Java-based software that enables visual exploration and knowledge mapping for bibliographic analysis. Through knowledge domain visualization, CiteSpace maps highly cited articles, crucial fields, and emerging topics in literature databases, revealing research trends and patterns [17]. Studies have shown that the Web of Science database exhibits a superior knowledge-mapping capability when integrated with CiteSpace for visual representation [18]. Therefore, selecting Web of Science as a data source is both reasonable and effective. For the visual representation of knowledge maps, we followed the fundamental procedures of CiteSpace, including time slicing, threshold setting, modeling, pruning, merging, and mapping. CiteSpace allows the analysis of citation bursts, betweenness centrality, and heterogeneous networks, facilitating the visualization of the current research status and hotspots and emerging research trends [15]. It integrates three functionalities, namely, bibliometrics, data integration, and visualization [19]. Basic bibliometric indicators, including countries, authors, institutions, keywords, and citations, can be analyzed using CiteSpace for the systematic visualization of research progress in a field of study and the prediction of future research directions [20]. In the authors’ analysis, the h-index is an essential metric. Introduced by physicist Jorge E. Hirsch in 2005 [14], the h-index evaluates both the quantity of a researcher’s publications and the frequency of their citations. This combined measure provides a comprehensive view of a researcher’s academic productivity and impact. In a particular analysis, nodes represent authors, institutions, countries, or keywords. The size of a node indicates the frequency of occurrence or citation, whereas its color represents the year of occurrence or citation. Furthermore, nodes edged in purple signify high centrality, typically indicating hotspots or pivotal points within the field [21]. The time slice was set from 2000 to 2023, and the included articles were visually analyzed based on countries/regions, authors, institutions, references, and keywords.

Pruning, a key feature of CiteSpace, improves the clarity of network visualization by minimizing link crossings. CiteSpace provides two pruning approaches: Pathfinder and Minimum Spanning Tree. According to Dr. Chaomei Chen, Pathfinder is more efficient in eliminating links. In particular, it can remove non-essential links while preserving critical ones [22]. Therefore, we selected Pathfinder for pruning.

## Results

### Quantitative analysis of basic information

#### Annual growth trend of publications

The trend of publication growth in a research field reflects the level of interest in the field and the importance of the field. A literature search in the WoSCC database revealed 4244 articles on the relationship between CRC and IBD published between January 1, 2000, and December 1, 2023. We excluded 5 duplicate articles using the duplicate remover feature in CiteSpace, eventually including 4239 articles for visual analysis. As shown in Figure S2 (Supplementary materials), research publications related to the relationship between IBD and CRC showed an overall trend of growth from 2000 to 2023. However, a slight decline was observed in 2001 (-2.78%), 2003 (-6.90%), 2004 (-6.17%), 2008 (-12%), 2011 (-7.95%), 2016 (-3.56%), and 2017 (-0.52%). On the contrary, the annual number of publications was higher in 2002 (24.29%), 2005 (21.05%), 2006 (20.65%), 2010 (33.33%), and 2019 (20.63%).

More than 100 articles were published annually after 2006 (Supplementary Table S1), with the fewest articles published in 2001 (*n* = 70, 1.65%) and the most articles published in 2022 (*n* = 344, 8.12%), averaging 177 articles per year. To predict the number of articles that would be published in 2024, a polynomial regression model was constructed using Microsoft Office Excel 365 (Microsoft, Redmond, WA, USA): y = 0.00574 × ^4^ − 0.2554 × ^3^ + 4.0265 × ^2^ − 14.999x + 87.965. A statistically significant correlation was observed between the year and the number of publications through data fitting (R^2^ = 0.9789). It is expected that 421 articles will be published in 2024.

#### Visualization analysis based on countries/regions

**Fig. 1:**
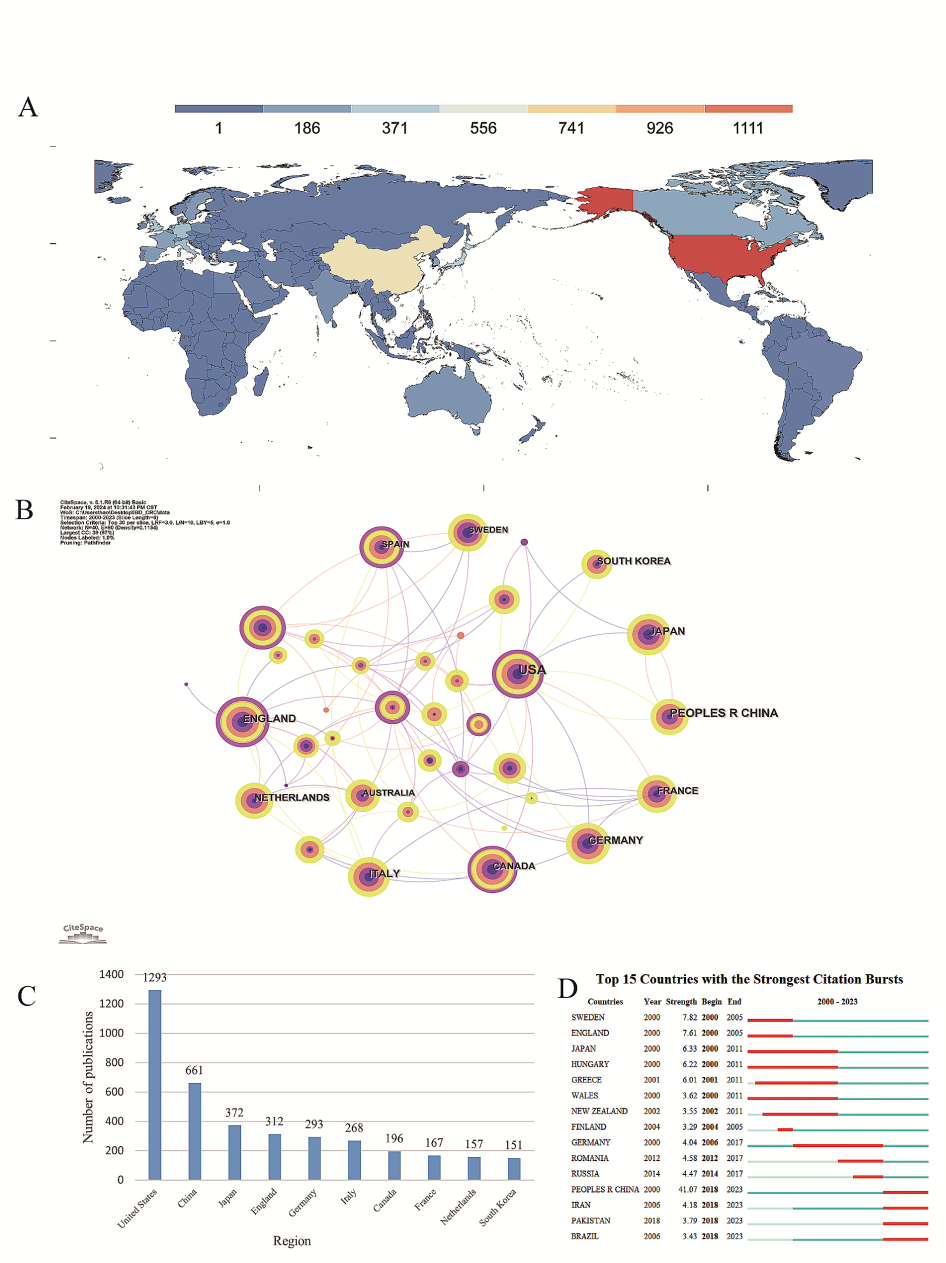
Visualization Analysis of Countries/Regions. (**A**) Distribution of countries in terms of publications. (**B**) Network diagram showing country links; time slice = 6, top 30 per slice; *N* = 40, E = 90. (**C**) Top 10 most productive countries. (**D**) Top 15 countries in publishing research on the association between IBD and CRC with burst period after 2000

Over the past 24 years, research articles on the relationship between IBD and CRC have been published in more than 100 countries/regions. To analyze the cooperation between countries and compare the number of publications, the time slice was set to 6 years per slice and the top 30 results from each slice were selected. As shown in the distribution map in Fig. 1A and the bar chart in Fig. 1C, the top 10 countries/regions (including 5 European countries, 3 Asian countries, and 2 North American countries) published a total of 3876 articles, accounting for 91.44% of the total publication volume. The five countries/regions with the highest number of publications were the United States of America (1293 articles, 30.50%), China (661 articles, 15.59%), Japan (372 articles, 8.78%), England (312 articles, 7.36%), and Germany (293 articles, 6.91%). The five countries with the highest centrality were Belgium (0.44), England (0.41), USA (0.28), Spain (0.22), and Denmark (0.22) (Table 1). Centrality values greater than 0.1 indicated a high level of influence in the research field. The USA not only led in publication volume but also had a centrality of 0.28, surpassing most countries/regions in terms of the quantity and quality of research articles on the relationship between IBD and CRC. Belgium had the highest centrality (0.44), demonstrating its prominent influence on the field and the high quality of research articles. Despite a low publication volume (14 articles), Greece had a high centrality of 0.22. Burst analysis (Fig. 1D) showed that research articles published from 2000 to 2011 were mainly concentrated in European countries/regions and Japan. Germany showed a significantly increased interest in the field from 2006 to 2017. After 2018, developing countries in Asia (such as China, Iran, and Pakistan) and South America (such as Brazil) showed a significantly increased interest in the field. In particular, China had the highest burst strength (41.07), suggesting that the relationship between CRC and IBD will remain a major research hotspot in China in the future. International collaboration was evident among the included countries/regions (Fig. 1B). Specifically, the USA collaborated with not just neighboring countries (Canada) but also European (UK, Norway, Belgium, and Greece) and Asian (Japan, China, and Korea) countries. On the contrary, China exhibited fewer collaborative efforts, focusing mainly on neighboring countries/regions (such as Japan) and the USA. In particular, it had a close partnership with Japan.

**Table 1:** Ranking of countries according to number of published articles and centrality

Rank	Country/region	Counts	Country/region	Centrality
1	USA	1293	BELGIUM	0.44
2	CHINA	661	ENGLAND	0.41
3	JAPAN	372	USA	0.28
4	ENGLAND	312	SPAIN	0.22
5	GERMANY	293	DENMARK	0.22

#### Visualization analysis based on research institutions

The cooperative and co-emerging networks of research institutions demonstrated the level of interdisciplinary research and collaborations between institutions. The time slice was set to 1 year per slice, and the top 10 research institutions from each slice were selected, with similar institutions (such as Harvard University and Harvard Medical School) being merged. The five institutions with the highest publication volume were Mayo Clinic (70), Harvard University (70), University of Washington (40), Cleveland Clinic (37), and University of Toronto (37) (Supplementary Table S2). With a centrality of 0.19, Mayo Clinic led in terms of both the number and quality of publications, profoundly influencing the research status of other institutions. Interestingly, Four of these five institutions are located in the United States of America.

**Fig. 2:**
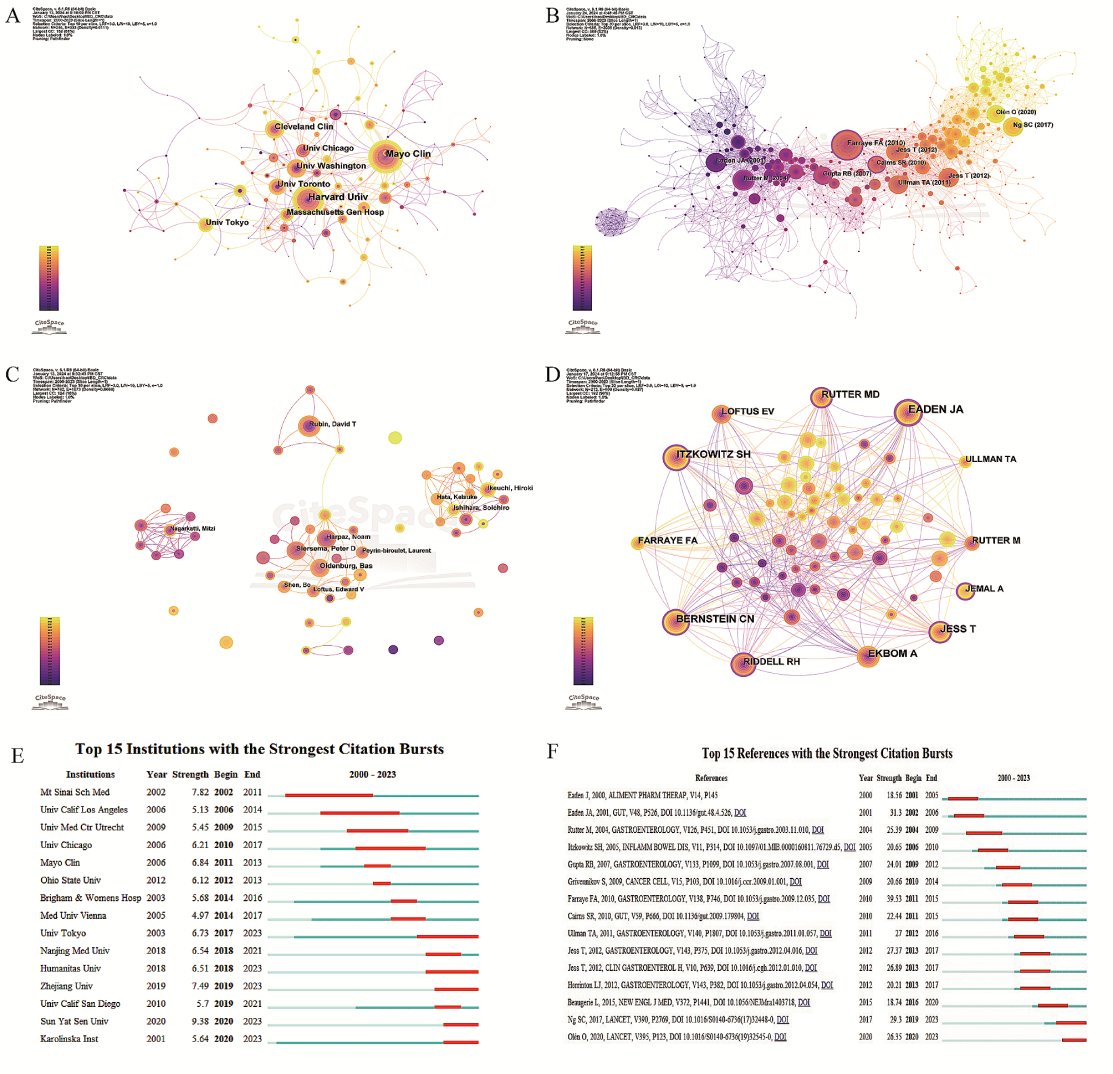
Visualization Analysis of Research Institutions, Authors and reference. (**A**) Network diagram showing institution links; Slice length = 1, Top 10 per slice, *N* = 246, E = 333. (**B**) Network diagram showing literature co-citations link; Slice length = 1; Top 30 per slice, *N* = 685, E = 3036. (**C**) Network diagram showing author links; Slice length = 1; Top 10 per slice, *N* = 742, E = 187. (**D**) Network diagram showing co-author links; Slice length = 1; Top 30 per slice, *N* = 213, E = 609. (**E**) Top 15 institutions in publishing research the association between IBD and CRC with burst period after 2000. (**F**) Top 15 articles related to research the association between IBD and CRC with burst period after 2000

Cooperation between institutions was more extensive and closer than that between countries (Fig. 2A). Mayo Clinic had the most collaborations with domestic and international institutions, such as University of Calif San Francisco, Shanghai Jiao Tong University, Uppsala University, Herlev University Hospital, University of Calif San Diego, Radcliffe Infirmary, and Icahn School of Medicine at Mt Sinai. With 32 articles and a centrality of > 0.1, Massachusetts General Hospital had close collaborations with many research institutions both domestically and internationally, indicating that strengthening communication and cooperation is an effective approach to producing excellent results.

Among Chinese research institutions, Shanghai Jiao Tong University had the highest number of publications and had close collaborations with foreign research institutions, including Karolinska Institutions, University of Calif San Francisco, University of Oxford, Uppsala University, Harvard University, Tongji University, Mayo Clinic, and Icahn School of Medicine at Mt Sinai, with the closest collaboration being with University of Oxford. Other Chinese universities, such as the University of Chinese Academy of Sciences and Nanjing Medical University, had a significant number of articles (more than 10) but rarely collaborated with foreign universities. The University of Chinese Academy of Sciences collaborated exclusively with domestic research institutions, such as Capital Medical University, Zhejiang University, and Chinese Academy of Sciences, with the closest collaboration being with Capital Medical University. Burst analysis (Fig. 2E) revealed that Mt. Sinai School of Medicine, University of California Los Angeles, and University Medical Center Utrecht were among the first institutions to recognize and comprehensively examine the relationship between IBD and CRC. The burst period for Mt. Sinai School of Medicine was longest, lasting until 2011, whereas that for Mayo Clinic was 2011–2013. The volume and quality of articles published by Mayo Clinic exceeded those of other institutions, highlighting the exceptional research prowess of the institution. In 2018, three of the six institutions experiencing a surge in research activity were from China, highlighting the increasing focus on the field of IBD and CRC within the Chinese scientific community. In particular, Sun Yat-sen University had the highest burst strength at 9.38, indicating its potential for conducting more influential research in the future.

#### Visualization analysis based on authors and co-cited authors

Author collaboration analysis revealed the status of cooperation among core authors. The time slice was set to 1 year per slice, and the top 10 authors from each slice were selected. The distribution of the number of publications among these 10 authors is shown in Supplementary Table S3. Bas Oldenburg from the Netherlands UMC had the highest number of publications (17 articles, h-index = 13). His most impactful publication was an article published in *Gut* in April 2019 [23]. This article presented a 15-year multi-center, multi-national study that showed that patients with IBD who had two consecutive negative results on colonoscopy during the monitoring period had a lower risk of developing advanced tumors. Furthermore, the results showed four clusters based on authors who published more than five articles each: a cluster led by Hiroki Ikeuchi and Soichiro Ishihara, a cluster led by Bas Oldenburg, a cluster led by David T Rubin, and a cluster led by Mitzi Nagarkatti (Fig. 2C). Of these four clusters, the cluster led by Bas Oldenburg had the most notable research achievements, with three of the top five authors with high publication volume belonging to this cluster. Research among the four clusters was relatively independent, with rare or almost no collaboration. Timeline analysis showed that research in the cluster led by Hiroki Ikeuchi and Soichiro Ishihara had primarily emerged in recent years and yielded abundant results. An important concern was that all authors had a centrality of < 0.1, indicating limited mutual influence among them.

Author co-citation refers to the citation of at least one article each from two or more authors in one or multiple consecutive articles. Anonymous authors were not included in the co-citation analysis. The time slice was set to 1 year per slice, and the top 30 authors with high citation counts were selected from each time slice. Authors with a total citation count of > 30 were included in network visualization (Fig. 2D). The top five authors with high citation count and centrality are shown in Supplementary Table S4. Professor JA Eaden from the United Kingdom had the highest citation count and centrality, highlighting his significant achievements in the research field and the widespread acceptance of his research findings.

### Assessment of research hotspots and progress of the relationship between IDB and CRC based on co-cited articles

#### Analysis of total citations

Co-citation is defined as the simultaneous citation of two or more articles in a third article. Co-citation analysis is a vital tool in bibliometrics that aids scholars in discovering new research directions, understanding the relationship between different research topics, and assessing mutual influence and collaboration networks within the academic community [24]. The time slice was set to 1 year per slice, and the 30 most cited articles were selected from each slice to construct a co-citation network map (Fig. 2B). The top 5 articles with high citation counts are shown in Supplementary Table S5. The most cited article (82) was published by Professor FA Farraye in 2010 in *Gastroenterology*. This article provided a comprehensive review of the diagnosis and management of colorectal tumors in patients with IBD. It discussed the history of dysplasia and factors that increase the risk of CRC in patients with IBD, including the duration of the disease, anatomical extent of the disease, concurrent conditions such as primary sclerosing cholangitis, and family history of CRC. The findings specifically suggested that patients with IBD with prolonged disease duration and more extensive disease spread had a higher risk of CRC. Additionally, the article explored monitoring strategies, the role of chemopreventive agents, and the application of new imaging technologies in the management of dysplasia and CRC secondary to IBD [25]. The second most cited article (60) was published by JA Eaden in *Gut* in 2001. This article represented a meta-analysis of studies on the risk of CRC in patients with UC. In particular, it analyzed 116 studies involving 54,478 patients. The results showed that the overall prevalence of CRC was 3.7% and 5.4% among patients with UC and pancolitis, respectively. The cumulative risk of CRC increased with the duration of UC or pancolitis: 2% at 10 years, 8% at 20 years, and 18% at 30 years. Pediatric patients with UC had a higher risk of CRC than adult patients. In addition, geographic location affected the risk of CRC, with the risk being higher in the USA and UK than in Scandinavia and other countries [26]. The third most cited article was published by Professor SC Ng in *The Lancet* in 2017. Although this article was published in recent years, it had a high citation count, indicating its significant impact on the research field. This article assessed the global incidence and prevalence of IBD (CD and UC) in the 21st century. The results indicated that although the incidence rate of IBD has stabilized in Western countries, it is increasing in recently industrialized countries, such as Brazil. Europe and North America had the highest prevalence rate of IBD at > 0.3%. These findings emphasize the importance of the prevention and effective management of IBD [27]. Notably, of the top five most cited articles, three articles were published in *Gastroenterology*, highlighting its significant influence and widespread recognition as a leading academic journal in the field of research on the relationship between IBD and CRC. In the citation network, nodes with a centrality value of > 0.1 were considered key nodes. The article with the highest centrality (0.43) was published by RB Gupta in *Gastroenterology* in 2007. This article had a significant impact on subsequent research into the relationship between IBD and CRC. It presented a study on a group of patients with UC undergoing regular endoscopic monitoring for dysplasia. The results showed that the severity of inflammation was an independent risk factor for advanced colorectal tumors in patients with UC with a longer disease course [28].

#### Analysis of co-citation bursts

Burst analysis allows the identification of research hotspots or trends, facilitating the timely discovery of articles that may significantly influence and guide future research [29]. Based on the results of burst analysis, Fig. 2F shows the top 15 articles ranked based on the citation burst strength. The article with the highest citation burst strength was also the most cited article. The article with the second highest citation burst strength was also the second most cited article. The article with the third highest citation burst strength also had a high citation count and was published by Jess T in 2012. This article reported that although the overall risk of CRC in patients with UC was comparable to that in the general population, it was higher in pediatric patients with UC, patients with UC with a longer disease course, or patients with UC with primary sclerosing cholangitis [30]. Based on the year of citation bursts, an article titled “CRC prevention in ulcerative colitis: a case-control study” was cited extensively in 2000. This article reported that regular treatment with 5-ASA significantly reduced the risk of CRC in patients with UC. Specifically, the use of mesalazine reduced the risk of CRC by 81%, a protective effect that was statistically significant at daily doses of ≥ 1.2 g. The protective effects of sulfasalazine were less pronounced and significant only at higher doses (≥ 2 g/day) [31]. A recent article titled “CRC in ulcerative colitis: a Scandinavian population-based cohort study” published in *The Lancet* had a high citation burst. This study compared the risk of CRC between 96,447 patients with UC and 949,207 control individuals from 1969 to 2017. The results showed that the incidence and mortality rates of CRC in patients with UC were higher than those in control individuals. Despite having early-stage tumors, patients with UC had a higher risk of CRC-related mortality. However, this risk decreased over time owing to improvements in treatment methods. These findings emphasize the necessity of improving international monitoring guidelines for CRC [32].

On analyzing articles with citation bursts, we observed a specific trend. Most articles suggested that patients with IBD, especially those with a longer disease course, had an increased risk of CRC. These articles included studies from various regions and with different populations, further validating the relationship between IBD and the incidence of CRC.

In the context of prolonged inflammatory responses, the mechanisms underlying CRC development are related to abnormalities in cellular signaling pathways, DNA damage, and disruption of DNA repair pathways. This finding holds substantial significance for clinical monitoring and treatment of IBD, indicating that physicians should increase the focus on CRC risk in patients with IBD with a prolonged disease course.

#### Analysis of the characteristics of co-citation clustering

**Fig. 3:**
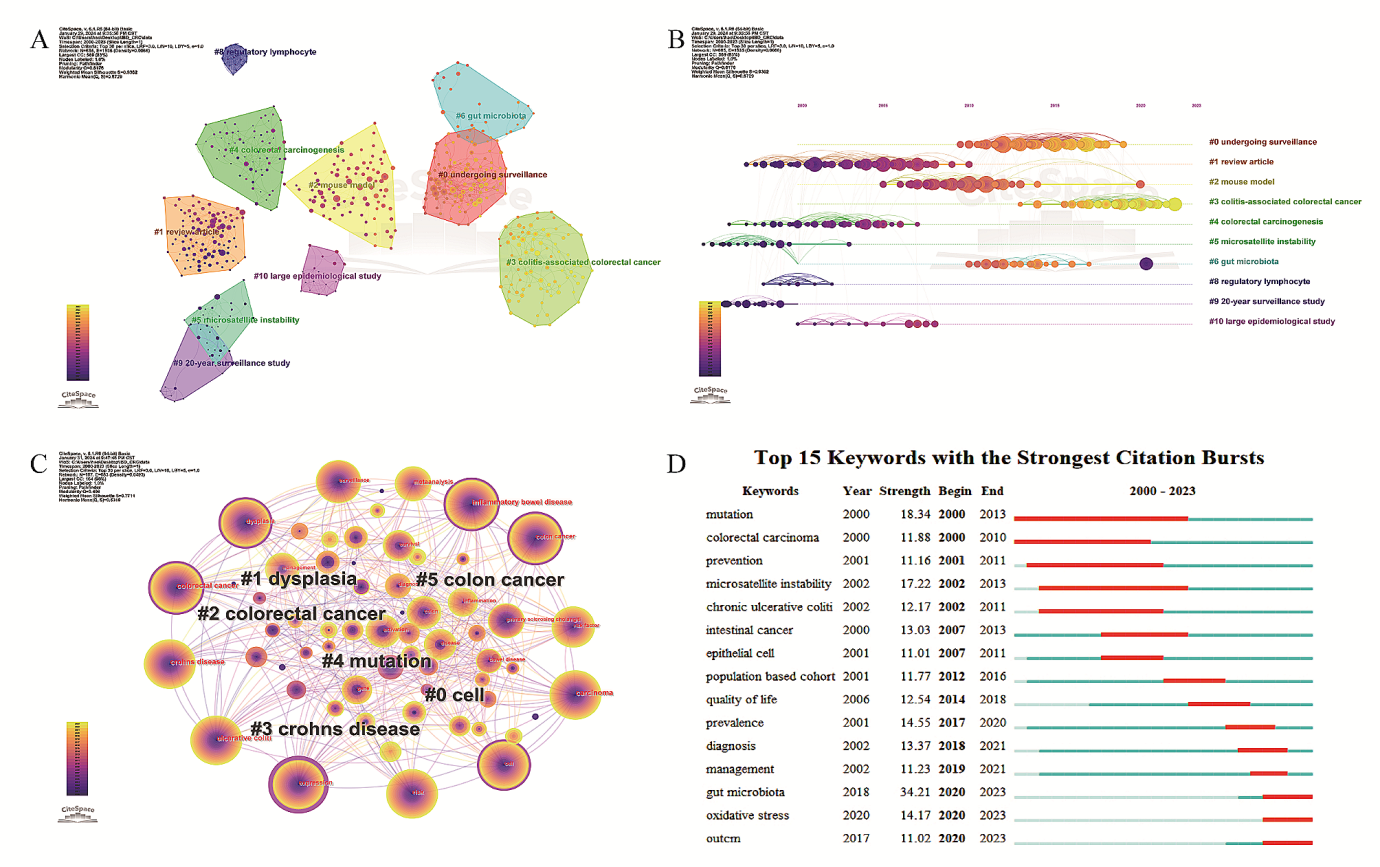
Analysis of the characteristics of Co-citation clustering and Keywords (**A**) Co-cited References Clustering Diagram. (**B**) Co-cited References Clustering Timeline Diagram. (**C**) Keyword Co-occurrence Network Diagram and Keyword Clustering. (**D**) Top 15 keywords related to research the association between IBD and CRC with burst period after 2000

The clustering function of CiteSpace was used to categorize research themes and exclude outliers. Cluster analysis of articles with citation bursts showed that research themes had undergone significant changes over the past 24 years. In Fig. 3A, lighter shades of yellow indicate periods closer to the current year. The silhouette coefficient “S” was used to evaluate the appropriateness of clustering. S values of > 0.5 indicated reasonable clustering, whereas S values of > 0.7 indicated convincing clustering. The silhouette coefficients of all clusters shown in Fig. 3A were > 0.8 (Supplementary Table S6). The primary research theme among earlier articles included microsatellite instability (MSI), a molecular marker of defective mismatch repair (MMR) that is present in approximately 15% of CRC cases and is more common in early stages than in advanced stages [33]. The molecular characteristics of inflammation-related CRC include alterations in p53, MSI, and lack of MUC5AC expression. These mechanisms are different from the typical and serrated mechanisms underlying the pathogenesis of CRC [34]. A significant increase was observed in the number of studies and review articles in the field of disease surveillance. A study involving a subset of the British population showed that the incidence of synchronous and metachronous tumors in patients with IBD was twice that in patients without IBD, with the difference being statistically significant. IBD-related CRC often occurs in younger patients, with the prognosis being worse than that of sporadic CRC. Consequently, identifying the causes of these differences may facilitate the development of more effective screening, monitoring, and treatment strategies for CRC and its precursors in high-risk populations [35]. Over the past few years, the focus of research has gradually shifted toward animal disease models, particularly mouse models, facilitating the discovery of new drugs for treating IBD-related CRC. For instance, a study showed that oral administration of GDNPs2 in mouse models of colitis alleviated acute colitis, enhanced intestinal repair, and prevented chronic colitis and colitis-associated cancer. In addition, oral administration of GDNPs2 improved the survival and proliferation of IECs, reduced the levels of pro-inflammatory cytokines (TNF-α, IL-6, and IL-1β), and increased the levels of anti-inflammatory cytokines (IL-10 and IL-22) in mouse models of colitis. These results indicate that GDNPs2 can mitigate damaging factors while promoting healing [36]. At present, pathological mechanisms involving the gut microbiome are receiving increasing attention in research on IBD and CRC, with several studies suggesting that IBD is a significant risk factor for CRC.

The co-citation network constructed using temporal nodes generated a timeline graph (Fig. 3B). The size of circles in this graph indicated the number of citations: the larger the circle, the higher the number of citations. The timeline graph revealed influential research findings in each period. Articles with the highest, second highest, and third highest citation counts were published in 2010, 2001, and 2017, respectively. This finding highlights the continuous emergence of high-quality research articles, validating the importance of the relationship between IBD and CRC. The cluster with the largest title was “#0 undergoing surveillance”, suggesting that regular endoscopic monitoring can effectively prevent the risk of progression to CRC in patients with IBD. A study showed that the incidence rate of CRC among patients with IBD undergoing regular surveillance was lower, with only 17 CRC cases identified in a follow-up assessment of 6823 cases. Most CRC cases (70%) could be attributed to inadequate surveillance conditions before the diagnosis, such as insufficient bowel preparation, inadequate surveillance intervals, or improper dysplasia management. These findings indicate that strict adherence to guidelines on disease surveillance may reduce the incidence of CRC [37]. The cluster with the largest silhouette coefficient was “#8 regulatory lymphocyte”. Articles in this cluster were similar to each other, showing a strong consistency in research topics or methods. In addition, these articles had good differentiation when compared with articles in other clusters, indicating uniqueness in research themes or directions. Given that regulatory lymphocytes are dysregulated in IBD and CRC, Treg-targeted therapies may be effective in treating or preventing these diseases [38]. Furthermore, previous studies on the relationship between IBD and CRC were concentrated in four clusters as follows: #5 microsatellite instability, #9 20-year surveillance study, #8 regulatory lymphocyte, and #1 review article. Notably, the clusters #6 gut microbiota and #3 colitis-associated CRC included articles published in recent years, representing the current research directions in the field of IBD and CRC.

### Assessment of research hotspot and progress of the relationship between IBD and CRC based on keywords

#### Analysis of keyword co-occurrence

Keywords are the essence of a research article. Analyzing keywords helps in summarizing research themes in a particular field, identifying research hotspots, and predicting future research trends. The time slice was set to 1 year per slice, and the top 30 keywords with the highest frequency in each time slice were selected. After similar keywords (e.g., cancer and carcinoma) were merged, a keyword co-occurrence graph was generated using a Pathfinder network (Fig. 3C). The top 10 keywords with the highest frequency are shown in Table 2. As disease names, the keywords “CRC”, “IBD”, “ulcerative colitis”, “carcinoma”, “Crohn’s disease”, and “colon cancer” had the highest frequency. Keywords such as “risk”, “expression”, “dysplasia”, and “meta-analysis” highlighted the core focus of research into the relationship between IBD and CRC over the past 24 years. Among the top 10 keywords with centrality values of > 0.1, the keywords other than disease names were “expression” and “dysplasia”. Some studies have indicated that certain genes are aberrantly expressed in inflammation-related CRC. Compared with sporadic CRC (sCRC), IBD-related CRC exhibits strong downregulation of negative regulators of WNT, namely, AXIN2 and RNF43, and a decreased frequency of copy number amplification in HNF4A (a negative regulator of WNT-induced epithelial–mesenchymal transition [EMT]) [39]. During the progression from UC to CRC, the downregulation of miR-615-5p may induce the upregulation of STC1, promoting tumor development and progression [40]. IBD-induced carcinogenesis is closely related to the recurrent inflammation and repair of the intestinal mucosa. In particular, intestinal inflammation leads to dysplasia, which eventually leads to CRC. In IBD, large mucosal areas with long-term inflammation are susceptible to tumorigenic transformation, a process referred to as “field cancerization” [41]. In the keyword co-occurrence network, primary sclerosing cholangitis (PSC) appeared frequently, with a centrality of 0.1. PSC is a progressive, cholestatic, inflammatory, fibrotic disease closely associated with IBD. Patients with PSC have an increased risk of malignant tumors [42]. In patients with PSC, abnormal bile acid metabolism leads to oxidative stress and toxic damage to intestinal cells, triggering the activation of intracellular inflammatory pathways and amplification of cell proliferation, consequently increasing the risk of malignant tumors [43].

**Table 2:** Ranking of Co-occurrence of keywords

Rank	counts	centrality	year	keywords
1	1496	0.14	2000	colorectal cancer
2	1413	0.19	2000	inflammatory bowel disease
3	1388	0.09	2000	ulcerative colitis
4	803	0.07	2000	carcinoma
5	726	0.07	2000	crohn’s disease
6	550	0.06	2000	risk
7	522	0.16	2000	colon cancer
8	497	0.28	2000	expression
9	282	0.15	2000	dysplasia
10	249	0.02	2007	metanalysis

#### Analysis of keyword bursts

Keyword burst analysis not only reflects changes in research hotspots in a field but also predicts future research trends. As shown in Fig. 3D, changes in keyword bursts over the past 24 years demonstrated the evolution of research hotspots in articles on the relationship between IBD and CRC. “Mutation” was the keyword with the earliest burst, the longest burst duration, and a high burst intensity (18.34). This finding indicates that researchers have been aware of the crucial role of genetic mutations in the progression from IBD to CRC for a long time. Compared with APC mutations, p53 mutations occur earlier in CAC(Colitis Associated Cancer), potentially representing a key initiating event [44]. Furthermore, APC mutations occur later than is typical in sporadic CRC [45]. In articles published before 2010, another keyword with a high burst strength (17.22) was microsatellite instability. The two most common somatic genetic abnormalities in CRC are classified as chromosomal instability (CIN) and microsatellite instability (MSI). These abnormalities occur at the same frequency in UC-related CRC and sporadic CRC; however, their timing and frequency are different in the dysplasia-carcinoma sequence associated with UC [4]. After 2010, the keyword “population-based cohort” began to experience a burst, indicating an increased focus on meta-analyses of population-based cohort studies. Since 2020, the frequency of the keywords “gut microbiota”, “oxidative stress”, and “outcome” has been increasing in research articles on the relationship between IBD and CRC. “Gut microbiota” had the highest burst strength (34.21), indicating that it is a current research hotspot in the field, attracting substantial research interest and resources. Numerous studies have shown that bacteria such as *Escherichia coli*, ETBF, and *Fusobacterium* are involved in chronic inflammation and cancer development in patients with IBD. For instance, the enterotoxin produced by ETBF, namely, *Bacteroides fragilis* toxin, cleaves the cell adhesion molecule E-cadherin, a key component involved in metalloproteinase-mediated cell adhesion [46]. This toxin can trigger pro-inflammatory and pro-tumorigenic cysteine proteases in colonic epithelial cells, thereby recruiting polymorphonuclear myeloid-derived cells to promote the occurrence of colon cancer [47]. Additionally, chronic oxidative stress (OS) can lead to the oxidation of biomolecules (nucleic acids, lipids, and proteins) or the activation of inflammatory signaling pathways, resulting in the activation of various transcription factors or the dysregulation of gene and protein expression, which eventually leads to tumor development or cancer cell survival [48]. We speculate that gut microbiota and oxidative stress will continue to be among the major hotspots in research on IBD and CRC in the future.

#### Clustering of co-occurring keywords

Figure 3C demonstrates the main clusters of keywords. The size of the first six clusters was ≥ 10, and their silhouette coefficients were > 0.5, indicating that the clustering was reasonable. The silhouette coefficients of clusters #1-#5 were > 0.7, indicating that the clustering was convincing (Supplementary Table S7). Among the four largest cluster categories, “dysplasia” had the highest silhouette coefficient and an earlier average year of study. This finding indicates that dysplasia has been a focus of research on the relationship between IBD and CRC for a long time. With the improvement of healthcare management and the continuous development of endoscopy, screening and monitoring programs involving colonoscopy have been used to detect, identify, or remove dysplastic or CRC tissues. Dysplastic tissues can be removed through endoscopic resection. However, early colectomy should be recommended for patients with IBD with unresectable tumors to reduce the overall incidence and mortality of CRC [49].

## Discussion

Over the past 24 years, an increasing number of studies have focused on the relationship between IBD and CRC. In this bibliometric study, we systematically searched the WoSCC database for articles on the relationship between IBD and CRC published in English between 2000 and 2023. After excluding duplicate articles and articles that did not meet the selection criteria, we eventually included 4239 research articles from 98 countries. The CiteSpace software was used for the quantitative analysis and visual representation of research progress to identify research hotspots and future research trends.

The number of research articles on the relationship between IBD and CRC increased annually over the past 24 years. The quadratic curve fit of cumulative publications suggested ongoing growth in research on IBD and CRC, reflecting an increasing interest in this research field within the scientific community. The United States of America had the highest publication volume, perhaps due to substantial NIH funding and a significant public health focus on IBD and CRC, driving extensive research and support for understanding, preventing, and treating these diseases. Although China ranked second in terms of publication volume, its centrality score was relatively low, suggesting that the quality and influence of articles published by Chinese researchers need improvement. The Mayo Clinic surpassed other institutions in terms of both publication volume and research quality owing to its robust research prowess and extensive collaboration network. Of the six research institutions that emerged in 2018, three were Chinese, indicating that the relationship between IBD and CRC is becoming a research hotspot in China.

Professor Bas Oldenburg from the Netherlands had the highest number of publications, demonstrating his in-depth research in the field. Professor JA Eaden from the UK had the highest number of co-citations, highlighting his significant influence on research into IBD and CRC. The most cited article (82 citations) was published by Professor FA Farraye in *Gastroenterology* in 2010. This indicates that the article is of high quality, with its theories, methods, or findings making significant contributions to academic research. It has garnered widespread attention and recognition, significantly impacting subsequent research. Cluster analysis indicated that earlier studies focused more on MSI to investigate the progression of IBD to CRC from a genetic perspective. IBD was consistently validated as a risk factor for CRC in numerous long-term, multicenter, large-sample surveillance studies. In recent years, the research focus has shifted toward the role of the gut microbiome in the progression of IBD to CRC. The largest cluster titled “undergoing surveillance” emphasized the important role of regular endoscopic monitoring in effectively preventing the development of CRC in patients with IBD.

Keyword co-occurrence and burst analysis revealed the main research themes and emerging research trends pertaining to the relationship between IBD and CRC. Earlier studies primarily focused on genetic mutations and MSI to investigate the pivotal roles of specific genes in the progression of IBD to dysplasia and CRC. Chronic inflammation-induced oxidative stress can lead to DNA damage, subsequently triggering the activation of oncogenes and the inactivation of tumor suppressor genes. Defects in the DNA mismatch repair mechanism result in high microsatellite instability (MSI-H), consequently promoting the initiation and development of CRC [50]. In recent years, gut microbiota and oxidative stress have become major hotspots in research on the relationship between IBD and CRC. The gut microbiome plays a key role in the development of IBD and CRC by regulating intestinal mucosal homeostasis, the intestinal microenvironment, and mucosal immunity. The gut microbiome comprises both probiotics that promote intestinal health and pathogens that cause inflammation and damage the mucosa. It serves as the first line of defense against the invasion of external pathogens; however, it can also stimulate inflammation and produce bacterial toxins that increase the risk of cancer. When the balance between probiotics and pathogens is disrupted, it destroys the normal intestinal homeostasis, leading to mucosal damage, a process referred to as “gut microbiota dysbiosis.” The development of IBD and CRC is closely related to the imbalance of the gut microbiome. In particular, the overgrowth of pathogens leading to inflammation can alter the structure of the gut microbiome and affect intestinal barrier function [51]. Under physiological conditions, a balance exists between the production of ROS and their elimination by antioxidants. However, under pathological conditions, such as chronic inflammation in IBD, the imbalance between ROS production and antioxidant defenses leads to oxidative stress, thereby exacerbating intestinal mucosal damage and inflammatory responses [52]. Oxidative stress promotes the progression of IBD and intestinal carcinogenesis by damaging cellular DNA and facilitating molecular events that lead to tumorigenesis.

In keyword clustering analysis, “dysplasia” had the largest silhouette coefficient and an earlier average year of study. Markers of oxidative damage and DNA double-strand breaks are gradually upregulated during the progression of inflammation to dysplasia and, eventually, cancer [53]. The natural history of dysplasia in colitis progresses from the absence of dysplasia in the intestinal mucosa to indefinite dysplasia, low-grade dysplasia (LGD), high-grade dysplasia (HGD), and invasive cancer. Factors leading to tumorigenesis in IBD can be summarized as follows: (1) genetic alterations (such as chromosomal alterations, MSI, and hypermethylation), (2) mucosal inflammatory mediators, (3) changes in the expression of receptors on epithelial cells, and (4) oxidative stress and gut microbiota [54]. Endoscopic monitoring of dysplasia is one of the important methods for preventing CRC in patients with IBD. According to the guidelines established by the Surveillance for Colorectal Endoscopic Neoplasia Detection and Management in Inflammatory Bowel Disease Patients (SCENIC), chromoendoscopy for colonoscopy is the optimal endoscopic strategy for identifying dysplasia. Procedures such as endoscopic mucosal resection (EMR) and endoscopic submucosal dissection (ESD) represent various treatment options for colonic dysplastic lesions in patients with IBD [55].

To the best of our knowledge, this study is the first to conduct a bibliometric analysis of research articles on the relationship between IBD and CRC. We obtained research articles published between 2000 and 2023 from the WoSCC database and conducted an objective and thorough analysis of these articles using CiteSpace. Despite its strengths, this study has some limitations that should be acknowledged. Only original articles published over the past 24 years were selected from the WoSCC database, excluding books, conference abstracts, and other types of articles. Therefore, this literature review may not cover every relevant article published to date. Limiting our review to articles published in English might have introduced potential language bias. Recent publications, particularly those of high quality, might have been underrepresented, emphasizing the requirement for continuous updates in this field. Although our dataset may not encapsulate the entirety of research in this domain, it is comprehensive enough to identify key research trends and focal points.

## Conclusion

This bibliometric study shows that the United States of America leads in research pertaining to the relationship between IBD and CRC, whereas China, Japan, and other countries have made significant research progress in this field. At present, gut microbiome and oxidative stress are major focus areas of research on the relationship between IBD and CRC. IBD has been validated as a risk factor for CRC. IBD-related CRC can be prevented through drug therapy (such as 5-ASA), regular endoscopic monitoring, and prompt endoscopic resection of dysplastic mucosal tissue. Future pre-clinical and clinical studies should adopt stricter research methodologies to develop more effective prevention and therapeutic strategies for IBD-related CRC.

## Significance and limitations

Significance: 1.This work marks the inaugural bibliometric exploration of the link between IBD and CRC, to the best of our understanding. 2. We sourced our dataset from the WoSCC database, capturing an extensive collection of articles focused on the IBD-CRC correlation. 3. Our methodology ensures an objective and thorough overview of the existing research landscape on this topic.

Limitations: (1) Our analysis spans original articles from the WoSCC database, dated between 2000 and 2023, excluding books, conference abstracts, and other formats from our selection process means our literature review may not cover every relevant work. (2) Limiting our review to English-language articles introduces a potential for language bias. (3) Our study focuses solely on WoSCC, potentially missing relevant research indexed in other databases, which might limit the comprehensiveness of our analysis.

### Electronic supplementary material

Below is the link to the electronic supplementary material.

Supplementary Material 1

## Data Availability

No datasets were generated or analysed during the current study.
