# Supplementary material for: Global research trends on the relationship between IBD and CRC: a bibliometric analysis from 2000 to 2023
Source: J Health Popul Nutr. 2024 Jun 12;43:83. doi: 10.1186/s41043-024-00577-5 (PMC11170923; doi:10.1186/s41043-024-00577-5)
Supplement: Supplementary file 1 — Supplementary Material 1 [file 41043_2024_577_MOESM1_ESM.docx]

**Literature search methods:**

Databases: SCI-EXPANDED, CPCI-S, CPCI-SSH, BKCI-S, BKCI-SSH

#1: TI= ("Inflammatory Bowel Disease") OR TI= ("IBD") OR TI= ("Idiopathic Proctocolitis") OR TI= ("UC") OR TI= ("Ulcerative Colitis") OR TI= ("Colitis Gravis") OR TI= ("Crohn's Enteritis ") OR TI= ("Regional Enteritis") OR TI= ("CD") OR TI= ("Crohn's Disease") OR TI= ("Granulomatous Enteritis") OR TI= ("Ileocolitis") OR TI= ("Granulomatous Colitis") OR TI= ("Terminal Ileitis") OR TI= ("Regional Ileitides") OR TI= ("Regional Ileitis") OR AB= ("Inflammatory Bowel Disease") OR AB= ("IBD") OR AB= ("Idiopathic Proctocolitis") OR AB= ("UC") OR AB= ("Ulcerative Colitis") OR AB= ("Colitis Gravis") OR AB= ("Crohn's Enteritis ") OR AB= ("Regional Enteritis") OR AB= ("CD") OR AB= ("Crohn's Disease") OR AB= ("Granulomatous Enteritis") OR AB= ("Ileocolitis") OR AB= ("Granulomatous Colitis") OR AB= ("Terminal Ileitis") OR AB= ("Regional Ileitides") OR AB= ("Regional Ileitis")

#2: TI= ("Colorectal Neoplasm") OR TI= ("CRC") OR TI= ("Colorectal Tumor") OR TI= ("Colorectal Cancer") OR TI= ("Colorectal Carcinoma") OR TI= ("Colonic Neoplasm") OR TI= ("COAD ") OR TI= ("Colon Neoplasm") OR TI= ("Colon Cancer") OR TI= ("Colonic Cancer") OR TI= ("Colon Adenocarcinoma") OR TI= ("Rectal Neoplasm") OR TI= ("READ") OR TI= ("Rectum Neoplasm") OR TI= ("Rectal Tumor") OR TI= ("Rectal Cancer") OR TI= ("Rectum Cancer") OR AB= ("Colorectal Neoplasm") OR AB= ("CRC") OR AB= ("Colorectal Tumor") OR AB= ("Colorectal Cancer") OR AB= ("Colorectal Carcinoma") OR AB= ("Colonic Neoplasm") OR AB= ("COAD ") OR AB= ("Colon Neoplasm") OR AB= ("Colon Cancer") OR AB= ("Colonic Cancer") OR AB= ("Colon Adenocarcinoma") OR AB= ("Rectal Neoplasm") OR AB= ("READ") OR AB= ("Rectum Neoplasm") OR AB= ("Rectal Tumor") OR AB= ("Rectal Cancer") OR AB= ("Rectum Cancer")

((#1 AND #2) AND DT=(Article)) AND LA=(English)

DOP: 2000-01-01 to 2023-12-01


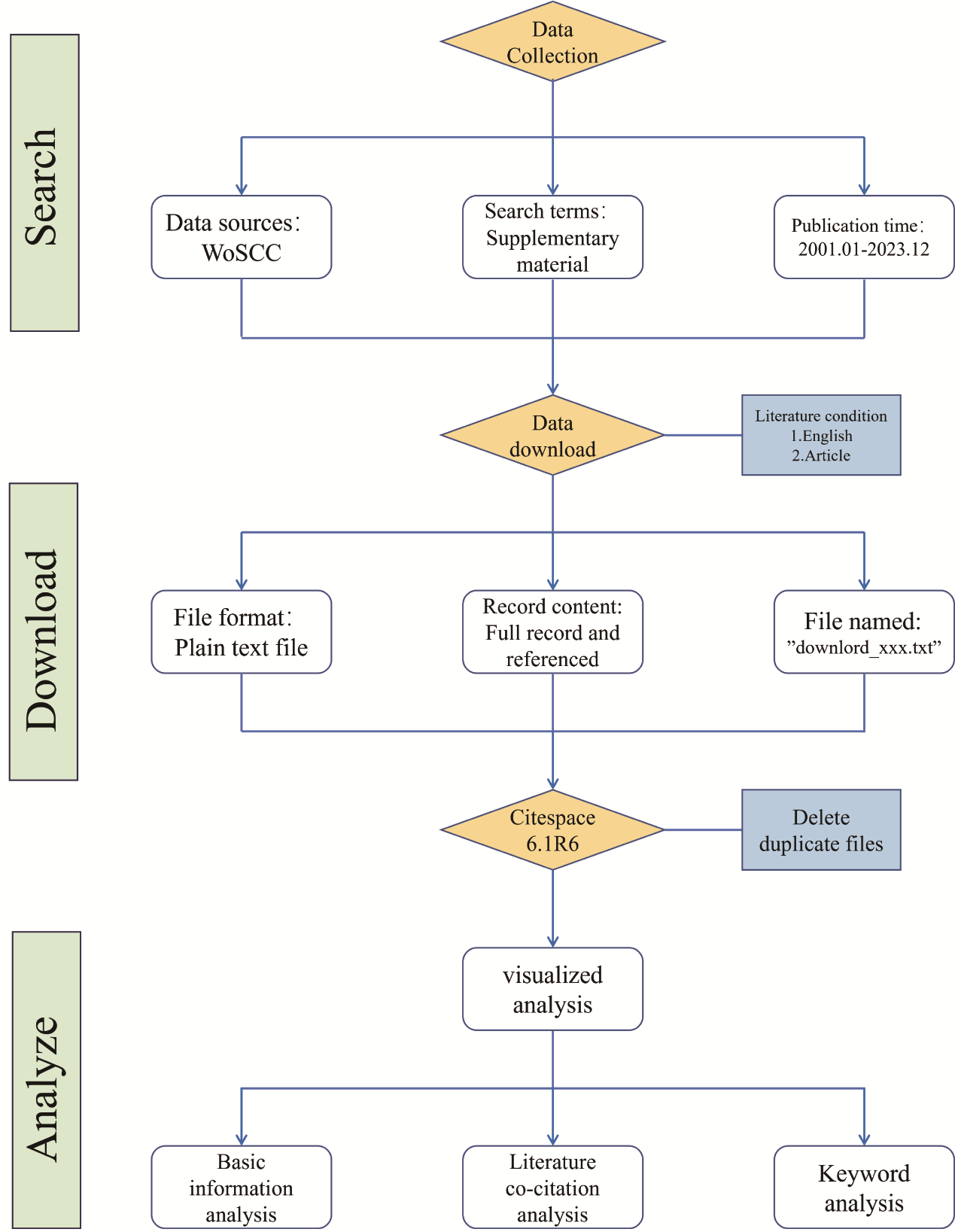


**Figure S1. Flowchart of the search process for included articles.**


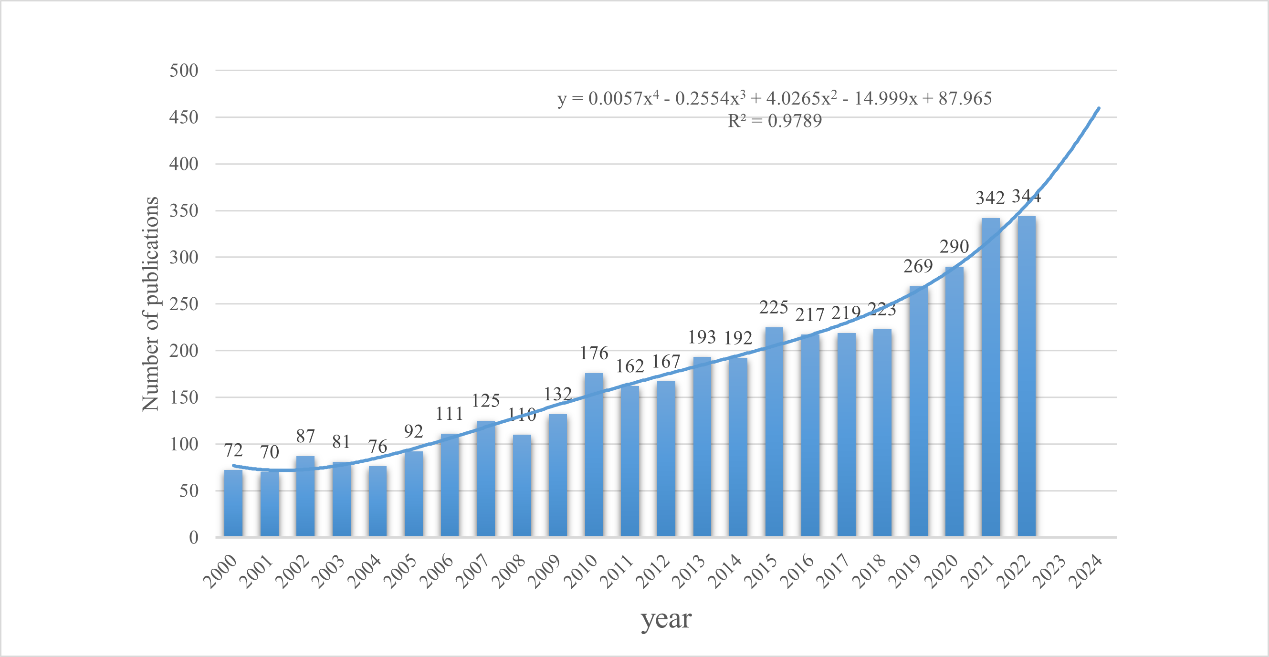


**Figure S2. Trend of numbers of publications on the relationship between IBD and CRC by years**

**Table S1 Annual growth rate of research on the relationship Between IBD and CRC**

| **Year** | **Publications** | **Annual growth rate(%)** |
| --- | --- | --- |
| 2000 | 72 |  |
| 2001 | 70 | -2.78 |
| 2002 | 87 | 24.29 |
| 2003 | 81 | -6.90 |
| 2004 | 76 | -6.17 |
| 2005 | 92 | 21.05 |
| 2006 | 111 | 20.65 |
| 2007 | 125 | 12.61 |
| 2008 | 110 | -12.00 |
| 2009 | 132 | 20.00 |
| 2010 | 176 | 33.33 |
| 2011 | 162 | -7.95 |
| 2012 | 167 | 3.09 |
| 2013 | 193 | 15.57 |
| 2014 | 192 | -0.52 |
| 2015 | 225 | 17.19 |
| 2016 | 217 | -3.56 |
| 2017 | 219 | 0.92 |
| 2018 | 223 | 1.83 |
| 2019 | 269 | 20.63 |
| 2020 | 290 | 7.81 |
| 2021 | 342 | 17.93 |
| 2022 | 344 | 0.58 |
| 2023(12/1) | 264 |  |

An average of 177 articles per year

**Table S2 Ranking of institutions according to number of published articles.**

| Rank | Counts | Centrality | Year | Institution |
| --- | --- | --- | --- | --- |
| 1 | 70 | 0.19 | 2006 | Mayo Clin |
| 2 | 70 | 0.09 | 2001 | Harvard Univ |
| 3 | 40 | 0.08 | 2001 | Univ Washington |
| 4 | 37 | 0.08 | 2008 | Cleveland Clin |
| 5 | 37 | 0.04 | 2001 | Univ Toronto |

**Table S3 Ranking of authors according to number of published articles.**

|  |  |  |
| --- | --- | --- |
|  |  |  |
|  |  |  |
|  |  |  |
|  |  |  |
|  |  |  |
|  |  |  |
|  |  |  |
|  |  |  |
|  |  |  |
|  |  |  |

| Rank | Author | Counts | h-index |
| --- | --- | --- | --- |
| 1 | Oldenburg, Bas | 17 | 13 |
| 2 | Siersema, Peter D | 16 | 13 |
| 3 | Rubin, David T | 15 | 14 |
| 4 | Harpaz, Noam | 14 | 13 |
| 5 | Ishihara, Soichiro | 14 | 6 |
| 6 | Ikeuchi, Hiroki | 14 | 8 |
| 7 | Loftus, Edward V | 12 | 11 |
| 8 | Peyrin-biroulet, Laurent | 11 | 11 |
| 9 | Hata, Keisuke | 11 | 7 |
| 10 | Shen, Bo | 11 | 6 |

**Table S4 Ranking of co-authors according to number of published articles and centrality**

| Rank | Co-cited author | Counts | Co-cited author | Centrality |
| --- | --- | --- | --- | --- |
| 1 | EADEN JA | 641 | EADEN JA | 0.22 |
| 2 | JESS T | 426 | RUTTER MD | 0.18 |
| 3 | EKBOM A | 403 | JESS T | 0.16 |
| 4 | BERNSTEIN CN | 375 | RIDDELL RH | 0.15 |
| 5 | ITZKOWITZ SH | 356 | BURMER GC | 0.13 |

**Table S5 Ranking of articles according to number of co-citation counts.**

| Rank | Counts | Centrality | Year | Co-cited reference |
| --- | --- | --- | --- | --- |
| 1 | 82 | 0.23 | 2010 | Farraye FA, 2010, GASTROENTEROLOGY, V138, P746, DOI 10.1053/j.gastro.2009.12.035 |
| 2 | 60 | 0.03 | 2001 | Eaden JA, 2001, GUT, V48, P526, DOI 10.1136/gut.48.4.526 |
| 3 | 59 | 0.02 | 2017 | Ng SC, 2017, LANCET, V390, P2769, DOI 10.1016/S0140-6736(17)32448-0 |
| 4 | 58 | 0.13 | 2011 | Ullman TA, 2011, GASTROENTEROLOGY, V140, P1807, DOI 10.1053/j.gastro.2011.01.057 |
| 5 | 58 | 0.03 | 2012 | Jess T, 2012, GASTROENTEROLOGY, V143, P375, DOI 10.1053/j.gastro.2012.04.016 |

**Table S6 Overview of main clusters cited in the articles.**

| **Cluster ID** | **Size** | **Silhouette** | **mean(Year)** | **Label (LLR)** |
| --- | --- | --- | --- | --- |
| 0 | 99 | 0.939 | 2014 | undergoing surveillance (610.4, 1.0E-4) |
| 1 | 96 | 0.892 | 2003 | review article (546.79, 1.0E-4) |
| 2 | 83 | 0.905 | 2009 | mouse model (564.19, 1.0E-4) |
| 3 | 83 | 0.954 | 2018 | colitis-associated colorectal cancer (725.68, 1.0E-4) |
| 4 | 70 | 0.926 | 2001 | colorectal carcinogenesis (421.92, 1.0E-4) |
| 5 | 30 | 0.938 | 1997 | microsatellite instability (181.79, 1.0E-4) |
| 6 | 29 | 0.984 | 2013 | gut microbiota (377.55, 1.0E-4) |
| 8 | 21 | 1 | 1999 | regulatory lymphocyte (16.98, 1.0E-4) |
| 9 | 19 | 0.982 | 1997 | 20-year surveillance study (117.81, 1.0E-4) |
| 10 | 17 | 0.997 | 2003 | large epidemiological study (92.62, 1.0E-4) |
| 11 | 9 | 0.995 | 1996 | e-cadherin gene (58.15, 1.0E-4) |
| 13 | 7 | 0.99 | 2006 | clinical trial (64.94, 1.0E-4) |
| 14 | 6 | 0.963 | 1998 | chronic ulcerative colitis-associated colorectal adenocarcinoma development (58.15, 1.0E-4) |

**Table S7 Overview of** **Keywords clustering.**

| **Cluster ID** | **Size** | **Silhouette** | **mean(Year)** | **Label (LLR)** |
| --- | --- | --- | --- | --- |
| 0 | 38 | 0.66 | 2007 | cell (83.78, 1.0E-4) |
| 1 | 36 | 0.805 | 2003 | dysplasia (58.55, 1.0E-4) |
| 2 | 23 | 0.783 | 2002 | colorectal cancer (30.03, 1.0E-4) |
| 3 | 21 | 0.78 | 2007 | crohns disease (109.67, 1.0E-4) |
| 4 | 18 | 0.76 | 2002 | mutation (25.37, 1.0E-4) |
| 5 | 13 | 0.825 | 2003 | colon cancer (159.15, 1.0E-4 |
| 6 | 9 | 0.948 | 2001 | thomsen-friedenreich antigen (22.16, 1.0E-4) |
| 7 | 6 | 0.848 | 2013 | quality of life (30.61, 1.0E-4) |
